# Supplementary material for: Hot spot prediction in protein-protein interactions by an ensemble system
Source: BMC Syst Biol. 2018 Dec 31;12(Suppl 9):132. doi: 10.1186/s12918-018-0665-8 (PMC6311905; doi:10.1186/s12918-018-0665-8)
Supplement: Supplementary file 2 — All relevant MATLAB code. A simple Matlab implement of our predictor. (ZIP 19,742 kb) [file 12918_2018_665_MOESM2_ESM.zip › Additional file 2/Read ASA/ASA website/BID/1DDMA.htm]

 NetSurfP - Protein Surface Accessibility and Secondary Structure Predictions

  

|  |  |  |
| --- | --- | --- |
|  |  | NetSurfP - Protein Surface Accessibility and Secondary Structure Predictions  Technical University of Denmark |

  


---

```
# For publication of results, please cite:
# A generic method for assignment of reliability scores applied to solvent accessibility predictions.
# Bent Petersen, Thomas Nordahl Petersen, Pernille Andersen, Morten Nielsen and Claus Lundegaard
# BMC Structural Biology 2009, 9:51 doi:10.1186/1472-6807-9-51
#
# Column 1: Class assignment - B for buried or E for Exposed - Threshold: 25% exposure, but not based on RSA
# Column 2: Amino acid
# Column 3: Sequence name
# Column 4: Amino acid number
# Column 5: Relative Surface Accessibility - RSA
# Column 6: Absolute Surface Accessibility
# Column 7: Z-fit score for RSA prediction
# Column 8: Probability for Alpha-Helix
# Column 9: Probability for Beta-strand
# Column 10: Probability for Coil
E H  1DDMA                  1    0.846 153.924   0.261   0.003   0.003   0.994
E Q  1DDMA                  2    0.628 112.214  -0.228   0.018   0.019   0.964
B W  1DDMA                  3    0.255  61.255  -1.505   0.018   0.019   0.964
E Q  1DDMA                  4    0.517  92.408  -0.107   0.018   0.047   0.935
E A  1DDMA                  5    0.305  33.600   0.253   0.018   0.019   0.964
E D  1DDMA                  6    0.586  84.399   1.285   0.053   0.005   0.942
E E  1DDMA                  7    0.601 105.082   0.979   0.694   0.003   0.303
E E  1DDMA                  8    0.673 117.486   0.448   0.694   0.003   0.303
E A  1DDMA                  9    0.403  44.455   1.434   0.622   0.015   0.363
B V  1DDMA                 10    0.084  12.957  -0.264   0.622   0.015   0.363
E R  1DDMA                 11    0.381  87.363   0.672   0.522   0.016   0.462
E S  1DDMA                 12    0.678  79.426   0.208   0.181   0.016   0.803
E A  1DDMA                 13    0.665  73.239  -0.724   0.018   0.019   0.964
E T  1DDMA                 14    0.398  55.244  -0.685   0.004   0.138   0.858
B C  1DDMA                 15    0.113  15.837  -0.201   0.004   0.616   0.381
E S  1DDMA                 16    0.295  34.562   0.747   0.001   0.900   0.099
B F  1DDMA                 17    0.073  14.671   1.122   0.001   0.959   0.040
E S  1DDMA                 18    0.324  37.985   1.503   0.001   0.959   0.040
B V  1DDMA                 19    0.023   3.458   0.972   0.001   0.959   0.040
E K  1DDMA                 20    0.307  63.150   1.509   0.001   0.959   0.040
B Y  1DDMA                 21    0.111  23.699   0.498   0.001   0.959   0.040
B L  1DDMA                 22    0.108  19.793   0.699   0.003   0.718   0.279
B G  1DDMA                 23    0.092   7.217  -1.636   0.022   0.552   0.426
B C  1DDMA                 24    0.206  28.880  -0.556   0.023   0.655   0.322
B V  1DDMA                 25    0.147  22.548   0.868   0.018   0.846   0.136
E E  1DDMA                 26    0.370  64.639   0.091   0.021   0.756   0.223
B V  1DDMA                 27    0.045   6.993  -0.239   0.022   0.359   0.619
E F  1DDMA                 28    0.538 107.936  -0.392   0.019   0.141   0.840
E E  1DDMA                 29    0.518  90.547  -0.136   0.018   0.088   0.893
E S  1DDMA                 30    0.343  40.188  -1.632   0.018   0.047   0.935
E R  1DDMA                 31    0.536 122.652  -0.119   0.018   0.019   0.964
B G  1DDMA                 32    0.273  21.517  -0.629   0.176   0.004   0.820
E M  1DDMA                 33    0.471  94.267   0.396   0.923   0.002   0.076
E Q  1DDMA                 34    0.579 103.427   1.380   0.970   0.001   0.030
B V  1DDMA                 35    0.104  15.939   1.032   0.970   0.001   0.030
B C  1DDMA                 36    0.018   2.583   1.244   0.970   0.001   0.030
E E  1DDMA                 37    0.374  65.303   1.860   0.988   0.000   0.012
E E  1DDMA                 38    0.500  87.420   2.054   0.988   0.000   0.012
B A  1DDMA                 39    0.025   2.711   0.642   0.988   0.000   0.012
B L  1DDMA                 40    0.039   7.159  -0.110   0.970   0.001   0.030
E K  1DDMA                 41    0.480  98.674   1.845   0.988   0.000   0.012
E V  1DDMA                 42    0.383  58.867   2.385   0.988   0.000   0.012
B L  1DDMA                 43    0.036   6.592   0.430   0.970   0.001   0.030
E R  1DDMA                 44    0.304  69.524   0.992   0.970   0.001   0.030
E Q  1DDMA                 45    0.610 108.892   0.314   0.923   0.002   0.076
E S  1DDMA                 46    0.519  60.815   0.115   0.694   0.003   0.303
E R  1DDMA                 47    0.435  99.546  -0.457   0.176   0.004   0.820
E R  1DDMA                 48    0.664 152.056   0.481   0.053   0.005   0.942
E R  1DDMA                 49    0.686 157.117   0.695   0.018   0.019   0.964
E P  1DDMA                 50    0.300  42.570  -0.048   0.005   0.015   0.979
E V  1DDMA                 51    0.408  62.786   0.212   0.004   0.138   0.858
E R  1DDMA                 52    0.499 114.271   1.782   0.004   0.616   0.381
B G  1DDMA                 53    0.030   2.385   0.200   0.001   0.900   0.099
E L  1DDMA                 54    0.246  45.116   1.720   0.001   0.959   0.040
B L  1DDMA                 55    0.023   4.303   0.700   0.001   0.959   0.040
E H  1DDMA                 56    0.275  50.059   1.186   0.001   0.959   0.040
B V  1DDMA                 57    0.027   4.165   0.983   0.001   0.959   0.040
B S  1DDMA                 58    0.114  13.326   0.680   0.003   0.718   0.279
E G  1DDMA                 59    0.370  29.150  -0.456   0.004   0.085   0.910
E D  1DDMA                 60    0.537  77.396   0.301   0.005   0.015   0.979
B G  1DDMA                 61    0.164  12.883   0.002   0.004   0.514   0.481
B L  1DDMA                 62    0.032   5.804   0.284   0.001   0.900   0.099
E R  1DDMA                 63    0.297  67.921   1.653   0.001   0.959   0.040
B V  1DDMA                 64    0.044   6.747   0.185   0.001   0.959   0.040
B V  1DDMA                 65    0.161  24.761   0.631   0.001   0.959   0.040
B D  1DDMA                 66    0.178  25.707  -0.245   0.003   0.718   0.279
E D  1DDMA                 67    0.490  70.609  -0.314   0.019   0.141   0.840
E E  1DDMA                 68    0.717 125.173   0.096   0.018   0.047   0.935
E T  1DDMA                 69    0.477  66.118  -0.394   0.018   0.019   0.964
E K  1DDMA                 70    0.641 131.813   0.048   0.018   0.019   0.964
E G  1DDMA                 71    0.493  38.783  -0.018   0.021   0.279   0.699
E L  1DDMA                 72    0.264  48.338   0.708   0.023   0.655   0.322
B I  1DDMA                 73    0.257  47.564  -0.751   0.023   0.655   0.322
B V  1DDMA                 74    0.283  43.466   0.085   0.023   0.655   0.322
E D  1DDMA                 75    0.540  77.742  -0.493   0.022   0.552   0.426
B Q  1DDMA                 76    0.148  26.361   1.334   0.004   0.420   0.576
E T  1DDMA                 77    0.437  60.612   1.018   0.004   0.197   0.799
B I  1DDMA                 78    0.058  10.656   0.348   0.004   0.197   0.799
E E  1DDMA                 79    0.332  57.948   0.645   0.005   0.262   0.733
E K  1DDMA                 80    0.291  59.859   0.909   0.004   0.420   0.576
B V  1DDMA                 81    0.018   2.705   1.410   0.001   0.900   0.099
B S  1DDMA                 82    0.047   5.497   0.039   0.001   0.959   0.040
B F  1DDMA                 83    0.038   7.727  -0.005   0.001   0.959   0.040
B C  1DDMA                 84    0.023   3.159   0.598   0.001   0.900   0.099
B A  1DDMA                 85    0.028   3.130  -0.723   0.003   0.718   0.279
B P  1DDMA                 86    0.240  33.999  -0.294   0.004   0.420   0.576
B D  1DDMA                 87    0.201  28.935   0.084   0.005   0.045   0.951
E R  1DDMA                 88    0.516 118.095  -0.934   0.005   0.015   0.979
E N  1DDMA                 89    0.644  94.340  -0.348   0.018   0.019   0.964
E H  1DDMA                 90    0.428  77.890   0.097   0.018   0.047   0.935
E E  1DDMA                 91    0.326  56.987   0.598   0.004   0.197   0.799
E R  1DDMA                 92    0.258  59.013   0.827   0.003   0.718   0.279
B G  1DDMA                 93    0.039   3.038   0.484   0.001   0.900   0.099
B F  1DDMA                 94    0.018   3.613   1.264   0.001   0.959   0.040
B S  1DDMA                 95    0.015   1.699   0.704   0.001   0.959   0.040
B Y  1DDMA                 96    0.061  13.143   0.932   0.001   0.959   0.040
B I  1DDMA                 97    0.026   4.847   0.530   0.001   0.900   0.099
B C  1DDMA                 98    0.053   7.455   0.172   0.021   0.756   0.223
E R  1DDMA                 99    0.342  78.204   0.378   0.004   0.616   0.381
E D  1DDMA                100    0.317  45.752   0.357   0.005   0.262   0.733
E G  1DDMA                101    0.668  52.564   0.040   0.005   0.045   0.951
E T  1DDMA                102    0.679  94.191  -0.833   0.005   0.015   0.979
E T  1DDMA                103    0.641  88.893  -0.081   0.005   0.015   0.979
E R  1DDMA                104    0.528 120.843  -0.322   0.005   0.015   0.979
E R  1DDMA                105    0.456 104.493   1.526   0.005   0.336   0.660
B W  1DDMA                106    0.095  22.775   0.373   0.002   0.816   0.182
B M  1DDMA                107    0.129  25.913   1.391   0.001   0.959   0.040
B C  1DDMA                108    0.019   2.668   1.143   0.001   0.959   0.040
B H  1DDMA                109    0.041   7.422   0.675   0.001   0.959   0.040
B G  1DDMA                110    0.024   1.865   0.948   0.001   0.959   0.040
B F  1DDMA                111    0.029   5.800   0.435   0.001   0.959   0.040
B L  1DDMA                112    0.213  38.982   1.353   0.001   0.959   0.040
B A  1DDMA                113    0.030   3.284   0.369   0.003   0.606   0.390
E C  1DDMA                114    0.425  59.684   0.698   0.004   0.085   0.910
E K  1DDMA                115    0.590 121.281   0.926   0.600   0.003   0.397
E D  1DDMA                116    0.661  95.207   1.094   0.858   0.002   0.139
B S  1DDMA                117    0.221  25.913   0.515   0.858   0.002   0.139
B G  1DDMA                118    0.028   2.196  -0.549   0.923   0.002   0.076
E E  1DDMA                119    0.431  75.261   1.533   0.970   0.001   0.030
E R  1DDMA                120    0.374  85.623   2.056   0.970   0.001   0.030
B L  1DDMA                121    0.021   3.918   1.486   0.988   0.000   0.012
B S  1DDMA                122    0.042   4.899   0.578   0.988   0.000   0.012
E H  1DDMA                123    0.371  67.430   1.883   0.988   0.000   0.012
B A  1DDMA                124    0.052   5.708   0.973   0.988   0.000   0.012
B V  1DDMA                125    0.027   4.135   1.035   0.988   0.000   0.012
B G  1DDMA                126    0.139  10.916   0.390   0.988   0.000   0.012
E C  1DDMA                127    0.356  49.940   1.213   0.970   0.001   0.030
B A  1DDMA                128    0.045   4.904   0.070   0.988   0.000   0.012
B F  1DDMA                129    0.073  14.631   0.385   0.988   0.000   0.012
E A  1DDMA                130    0.445  49.050   1.867   0.970   0.001   0.030
E V  1DDMA                131    0.282  43.282   1.191   0.970   0.001   0.030
B C  1DDMA                132    0.160  22.450   0.388   0.923   0.002   0.076
E L  1DDMA                133    0.300  54.875   0.061   0.802   0.014   0.185
E E  1DDMA                134    0.649 113.310   1.511   0.522   0.016   0.462
E R  1DDMA                135    0.794 181.803   1.296   0.016   0.005   0.979

Explain the output.  Go back.
```

---
